# Book Reviews

**Published:** 1902-03

**Authors:** 


The Standard Medical Directory of North America, consisting of Twelve Parts, including Directory of Physicians of North America, Medical Colleges, Medical Service of the United States, Medical Societies, Medical Practice, Acts, Medical Publications (including Books and Periodicals), Mineral Springs, Drugs and Medicines, Medical and Surgical Products, Manufacturers and Life Insurance Companies. Handsomely bound in red buckram, 824 pages, imperial octavo. Price, $10. G. P. Englehard & Co., Chicago.

The arrangement of this directory is commendable, and it will probably prove of value; but the proof reading is so slovenly that the merit of the work almost is destroyed. This of course refers to the Atlanta list, in which many familiar names are distorted beyond recognition, while many others are omitted altogether. This is a bad error in any publication, in a directory it is unpardonable. A prompt revision seems to be the only remedy.

